# The role of suicide severity in the association between anxiety symptoms and suicidal ideation: a mediation analysis

**DOI:** 10.1192/j.eurpsy.2024.1627

**Published:** 2024-08-27

**Authors:** M. Cifrodelli, E. Rogante, A. Moschillo, L. Longhini, M. A. Trocchia, D. Erbuto, S. Sarubbi, I. Berardelli, M. Pompili, M. Innamorati

**Affiliations:** ^1^Psychiatry Unit, Sapienza University of Rome, Faculty of Medicine and Psychology, Psychiatry Residency Training Program, Via di Grottarossa, 1033-1035, 00189, Rome; ^2^Department of Human Neurosciences, Sapienza University of Rome, Viale Dell’università, 30, 00185, Rome; ^3^Department of Neurosciences, Mental Health and Sensory Organs, Faculty of Medicine and Psychology, Suicide Prevention Centre, Sapienza University of Rome, Via di Grottarossa, 1033-1035, 00189, Rome; ^4^Department of Human Sciences, European University of Rome, Via degli Aldobrandeschi, 190, 00163, Rome, Italy

## Abstract

**Introduction:**

Suicide is one of the leading causes of death worldwide and scientific community investigates suicide risk factors relentlessly. Among these, anxiety symptoms were strongly related to suicidal ideation in several studies. Moreover, sleep and its disturbances are closely connected to mental well-being and psychiatric disorders in a bidirectional pathway.

**Objectives:**

The main purpose of the present study is to assess the relationship between anxiety symptoms and suicidal ideation in a sample of psychiatric patients and the mediational role of insomnia in this association.

**Methods:**

Participants were 116 consecutive adult psychiatric inpatients (61 women and 55 men) enrolled to the psychiatric inpatient unit of Sant’Andrea Hospital in Rome. The measures used were a socio-anamnestic form, the Columbia Suicide Severity Rating Scale (C-SSRS), the Hamilton Anxiety Rating Scale (HAM-A), and the Insomnia Severity Index (ISI).

**Results:**

Based on the results of statistical analysis, patients with suicidal ideation showed higher severity of insomnia and higher severity of anxiety symptoms than patients with no suicidal ideation. Moreover, the intensity of suicidal ideation was positively and significantly associated with the severity of anxiety symptoms and with the severity of insomnia. Finally, the mediation analysis showed that the effect of anxiety symptoms on suicidal ideation was completely mediated by insomnia severity.

**Conclusions:**

The main result of the study indicates that patients who perceive more anxiety symptoms were more likely to experience higher suicidal ideation intensity through higher levels of insomnia. These findings implies that screening for sleep disturbances may help identify individuals at risk for suicide, and improving sleep quality through psychosocial and pharmacological treatments could mitigate the association between anxiety and suicidal ideation.

**Disclosure of Interest:**

None Declared

